# The facilitators and barriers associated with implementation of a patient-centered medical home in VHA

**DOI:** 10.1186/s13012-016-0386-6

**Published:** 2016-02-24

**Authors:** Christian D. Helfrich, Philip W. Sylling, Randall C. Gale, David C. Mohr, Susan E. Stockdale, Sandra Joos, Elizabeth J. Brown, David Grembowski, Steven M. Asch, Stephan D. Fihn, Karin M. Nelson, Lisa S. Meredith

**Affiliations:** 1 Seattle-Denver Center of Innovation for Veteran-Centered and Value-Driven Care, VA Puget Sound, U.S. Department of Veterans Affairs, 1660 Columbian Way, S-152, Seattle, 98108 WA USA; 2 Department of Health Services, University of Washington School of Public Health, Seattle, WA USA; 3 Office of Analytics and Business Intelligence, U.S. Department of Veterans Affairs, Seattle, WA USA; 4 Center for Innovation to Implementation, VHA Palo Alto Healthcare System, Menlo Park, CA USA; 5 Center for Healthcare Organization and Implementation Research (CHOIR), VA Boston Healthcare System, Boston, MA USA; 6 Boston University School of Public Health, Boston, MA USA; 7 HSR&D Center for the Study of Healthcare Innovation, Implementation, and Policy, VHA Greater Los Angeles Health Care System, North Hills, CA USA; 8 Department of Psychiatry and Biobehavioral Sciences, David Geffen School of Medicine, University of California, Los Angeles, CA USA; 9 Portland VHA Medical Center, VISN 20 Patient Aligned Care Team (PACT) Demonstration Laboratory, U.S. Department of Veterans Affairs, Portland, OR USA; 10 Center for Evaluation of Patient Aligned Care Teams (CEPACT), Philadelphia Veterans Affairs Medical Center, Philadelphia, USA; 11 The Robert Wood Johnson Foundation Clinical Scholars Program, and the Department of Family and Community Medicine, Perelman School of Medicine of the University of Pennsylvania, Philadelphia, USA; 12 Stanford University School of Medicine, Palo Alto, CA USA; 13 Department of Medicine, University of Washington School of Medicine, Seattle, WA USA; 14 RAND Corporation, Santa Monica, CA USA; 15 VA HSR&D Center for the Study of Healthcare Innovation, Implementation, and Policy, Los Angeles, CA USA

## Abstract

**Background:**

The patient-centered medical home (PCMH) is a team-based, comprehensive model of primary care. When effectively implemented, PCMH is associated with higher patient satisfaction, lower staff burnout, and lower hospitalization for ambulatory care-sensitive conditions. However, less is known about what factors contribute to (or hinder) PCMH implementation.

We explored the associations of specific facilitators and barriers reported by primary care employees with a previously validated, clinic-level measure of PCMH implementation, the Patient Aligned Care Team Implementation Progress Index (Pi^2^).

**Methods:**

We used a 2012 survey of primary care employees in the Veterans Health Administration to perform cross-sectional, respondent-level multinomial regressions. The dependent variable was the Pi^2^ categorized as high implementation (top decile, 54 clinics, 235 respondents), medium implementation (middle eight deciles, 547 clinics, 4537 respondents), and low implementation (lowest decile, 42 clinics, 297 respondents) among primary care clinics. The independent variables were ordinal survey items rating 19 barriers to patient-centered care and 10 facilitators of PCMH implementation. For facilitators, we explored clinic Pi^2^ score decile both as a function of respondent-reported availability of facilitators and of rating of facilitator helpfulness.

**Results:**

The availability of five facilitators was associated with higher odds of a respondent’s clinic’s Pi^2^ scores being in the highest versus lowest decile: teamlet huddles (OR = 3.91), measurement tools (OR = 3.47), regular team meetings (OR = 2.88), information systems (OR = 2.42), and disease registries (OR = 2.01). The helpfulness of four facilitators was associated with higher odds of a respondent’s clinic’s Pi^2^ scores being in the highest versus lowest decile. Six barriers were associated with significantly higher odds of a respondent’s clinic’s Pi^2^ scores being in the lowest versus highest decile, with the strongest associations for the difficulty recruiting and retaining providers (OR = 2.37) and non-provider clinicians (OR = 2.17). Results for medium versus low Pi^2^ score clinics were similar, with fewer, smaller significant associations, all in the expected direction.

**Conclusions:**

A number of specific barriers and facilitators were associated with PCMH implementation, notably recruitment and retention of clinicians, team huddles, and local education. These findings can guide future research, and may help healthcare policy makers and leaders decide where to focus attention and limited resources.

## Background

The patient-centered medical home (PCMH) is a model of primary care that seeks to change the current episodic, physician-centric model. PCMH is characterized by empaneled patients cared for by a provider-directed team; a whole-person orientation; emphasis on coordination of care, quality and safety; and enhanced access, such as via electronic and telephone communications [1]. While findings are mixed on whether the PCMH model improves outcomes such as quality of care, patient satisfaction and costs [1–4], the adoption of PCMH models is rapidly increasing among US primary care organizations. In a national survey of medical home initiatives involving payment reform, between 2009 and 2013, PCMH initiatives increased from 26 initiatives covering approximately 5 million patients to 114 initiatives covering approximately 21 million patients [5].

The factors that foster or hinder PCMH implementation are less clear despite the considerable investments of time, resources, and other support [6]. For example, organizational learning strategies, notably learning collaborative, and practice facilitation (practice-change experts providing on-site assistance in combination with other resources such as access to national consultants in practice economics, health IT, etc.), have been the dominant strategies used, but their effectiveness remains uncertain [4]. Qualitative studies have suggested successful implementation of PCMH requires the presence of engaged, visible leaders [7], who provide an explicit change strategy [8]; the creation of protected time for panel management; daily team huddles; and explicit work load credit for non-face-to-face modalities for delivering clinical care [9]. On the other hand, standard process improvement practices are infrequently used [10]. To close knowledge gaps about factors contributing to PCMH implementation, we explored the associations of specific barriers and facilitators with a validated measure of PCMH implementation in a large, integrated health delivery system implementing a PCMH model in more than 900 clinics.

## Methods

In 2010, the Veterans Health Administration (VHA) launched the Patient Aligned Care Teams (PACT) initiative to implement a PCMH model in all VHA primary care clinics simultaneously, over 900 clinics nationally. As part of efforts to evaluate the VHA PCMH initiative, extensive national data were collected in 2012 on clinic-level progress in implementing VHA PCMH and factors facilitating and impeding implementation. We assessed the associations between responses to an employee-based survey about VHA PCMH implementation and a validated measure of overall clinic-level VHA PCMH implementation.

### Measures and sample

Independent variables were obtained from a national, web-based survey fielded to all VHA primary care personnel in May and June 2012. The survey link was distributed via an email from VHA central office leadership to regional network and facility leaders with instructions to distribute to primary care personnel. Because the survey was anonymous and voluntary, the precise denominator is unknown; however, the estimated response rate was 25 %. Respondents to the VHA PCMH survey had very similar demographics with primary care respondents from a general employee survey that was fielded the month prior to the VHA PCMH survey. The general employee survey achieved a 62 % response rate. Tables comparing demographic variables have previously been published, and show that the PCMH sample had a slightly higher proportion of supervisors and slightly fewer African Americans but was not significantly different in terms of employee age, tenure, gender, or other racial or ethnic groups [11]. The present analysis focuses on the 96 clinics with survey responses (*n* = 532) from among the 164 clinics in the top and bottom deciles for VHA PCMH implementation, described further below. Analyses of middle decile clinics are included in the Appendices 1, 2, and 3. The survey methodology has previously been reported [11].

The VHA PCMH survey included items assessing barriers to the delivery of optimal patient-centered care and facilitators of PCMH implementation. The items were developed in collaboration with five regional Demonstration Laboratories, which were established to evaluate the impact of the PCMH initiative in VHA and test methods of improvement [12]. The survey also included respondent characteristics such as team role, proportion of time spent working in primary care, and supervisory level.

The independent variables were 19 items assessing the barriers and 10 items assessing the facilitators. The question stem for the 19 barrier items was, “How much, if at all, does each of the following factors limit your ability to provide optimal, patient-centered care for your patients?” The barrier items were preceded by a definition of patient-centered care, “An approach to healthcare that prioritizes the Veteran and their values and partners with them to create a personalized strategy to optimize their health, healing and well-being.” Respondents scored each barrier on a 3-point ordinal scale from “Does not limit,” to “Limits somewhat,” to “Limits a great deal.” There was also an “N/A or Don’t Know” response option. Barriers included factors such as difficulty accessing specialists, recruiting and retaining providers, and high-volume of Computerized Patient Record System (CPRS) automated alerts (e.g., for contraindicated medications).

The question stem for the 10 facilitator items was, “How helpful are the following [PCMH]-related activities or resources?” Respondents scored each facilitator on a four-point ordinal scale from “Very Helpful,” “Somewhat Helpful,” “Not Helpful,” to “Not Available/Not Involved.” They could also respond “N/A or Don’t Know.” Facilitators included local education sessions (e.g., a facility or team level in-service on PCMH to help define team roles and responsibilities of the different team members); measurement tools (e.g., use of patient data to evaluate improvement benchmarks); and information systems (e.g., the Primary Care Management Module is a suite of software tools that can be used by primary care teams to assign patients to teams and generate reports). The dependent variable was a validated composite measure of PCMH implementation, the Patient Aligned Care Team Implementation Progress Index (Pi^2^), which assesses progress in implementing PCMH within VHA, and incorporates data from surveys of primary care personnel, patient surveys, and administrative data [13]. The Pi^2^ includes 53 measures mapped to 8 domains representing goals of the initiative related to improving (1) access; (2) continuity; (3) care coordination; (4) comprehensive care; (5) self-management support; (6) patient-centered care and communication; (7) shared decision-making; and (8) team-based care. The Pi^2^ score is calculated as the number of domains in which the clinic appeared in the top and bottom quartiles for the domain scores, ranging from 8 (all domain scores in the top quartile) to −8 (all domain scores in the bottom quartile). The Pi^2^ has previously been validated by testing the association of clinics in the highest decile of the Pi^2^ (a score of +5 to +8) versus those in the lowest decile of the Pi^2^ (a score of −8 to −5) using measures of quality of care and outcomes that were expected to be favorably associated with PCMH implementation, including higher patient satisfaction, lower staff burnout, higher quality of care, and lower emergency department visits and hospitalizations for ambulatory care-sensitive conditions [13]. For the present analysis, we compared clinics in the lowest decile (−8 to −5, *n* = 87) to the highest decile (+5 to +8, *n* = 77); 96 of the 164 clinics in the high and low groups had one or more survey respondents.

Covariates included respondent characteristics, clinic workload and capacity, and setting. Respondent characteristics included VHA tenure, supervisory level, percentage of work hours spent in primary care teams, and occupation. Clinic workload and capacity included four variables from administrative data: (1) overall clinic average panel size, which was adjusted for type of provider (physician vs. nurse practitioner and physician assistant) and FTE; (2) the degree to which the clinic was over paneled relative to a 1200 patients per provider target panel size (0 if panel size ≤1200, the percent that average panel size exceeded 1200 if panel size >1200) [12]; and (3) clinical “intensity” of the patients in the panel, which was the average Diagnostic Cost Group score. Diagnostic Cost Group score is based on inpatient and outpatient diagnoses using the risk adjustment condition categories used by the Center for Medicare and Medicaid Services [14]. For setting, we adjusted for whether the clinic was community based or located with a VHA medical center, the logarithm of total annual visits, and whether the clinic had an academic-affiliation agreement indicating a teaching facility. The variables were selected because we expected they would reflect variation in local patient demand and workforce availability.

### Analyses

We conducted respondent-level multinomial logistic regressions, in which the dependent variable was the respondent’s clinic’s Pi^2^ score, categorized as high (top decile), medium (middle eight deciles), and low (lowest decile), as a function of barriers and facilitators. We ran separate models for each barrier and facilitator and adjusted for respondent characteristics and clinic-level workload and staffing measures in each model, described above.

For facilitators, we constructed two sets of models. In the first, we assessed the associations of facilitator “availability” with Pi^2^ score (high vs. low). A facilitator was defined as unavailable if the respondent rated it Not Available/Not Involved; otherwise, it was defined as available. We constructed a second set of models based upon respondents’ categorization of the facilitators as, Very Helpful versus Not or Somewhat helpful.

For primary analyses, responses that were NA/Don’t Know were categorized as missing and excluded from the relevant individual model using a standard listwise deletion approach, eliminating observations with missing values from a given model but including those observations in any models where values were not missing. Because NA/Don’t Know responses might actually indicate the absence of a barrier or facilitator (rather than truly missing data), we also conducted secondary analysis where NA/Don’t Know values were re-coded as a negative response rather than missing (i.e., not a barrier; facilitator not available; or facilitator not helpful). Because frequent NA/Don’t Know responses might be indicative of a respondent who either truly did not know or was passively filling out the survey, we also ran the secondary models excluding respondents with 70 % or more NA/Don’t Know on items.

In total, we constructed 39 models: 10 models on facilitators available/not available; 10 models on facilitator helpfulness; 19 models on barriers. For reporting purposes, in facilitator models, low implementation clinics were the referent and in barrier models, high implementation clinics were the referent. To simplify the presentation of data, we focus on findings comparing high versus low Pi^2^ clinics which are presented in the main results, but also include findings for comparisons of medium Pi^2^ clinics versus low (referent) for facilitators, and low Pi^2^ clinics versus medium (referent) for barriers in the appendices. Models were cluster-adjusted at the clinic level to allow for correlated responses within clinics. We report adjusted odds ratios and 95 % confidence intervals.

In order to assess potential non-response bias, we compared clinics without survey response to clinics with survey responses on covariates. We also assessed the level of inter-rater agreement on barriers and facilitators within site and within occupation.

Analyses were conducted using SAS Enterprise Guide 6.1.

## Results

The mean number of respondents per clinic was 5.5 respondents and the median was 3. Over 60 % of the respondents had been employed by VHA 5 years or longer. Forty-two percent (42 %) had some supervisory responsibilities and 88 % worked four-fifths or more of their time in primary care (Table 1). The respondents were approximately equally split among 3 of the 4 occupations: primary care provider (PCP), nurse care manager, or clinical associate, and 12 % of the respondents were administrative clerks.

**Table 1:** Respondent characteristics (*n* = 532, sites = 96)

Covariates	Proportion
Respondent characteristics	
Tenure with VA (%)	
Up to 1 year	7.5 %
Between 1 and 5 years	29.5 %
Between 5 and 10 years	22.9 %
Between 10 and 20 years	26.9 %
Over 20 years	13.2 %
Supervisory responsibilities	
None	58.3 %
Team Leader	34.3 %
Manager	2.8 %
First Line Supervisor	4.3 %
Executive/Senior Executive	0.4 %
Time spent in primary care	
<20 %	4.8 %
20–40 %	1.7 %
41–60 %	1.2 %
61–80 %	4.0 %
>80 %	88.2 %
Occupation (%)	
Primary Care Provider	31.1 %
Nurse Care Manager (RN)	32.4 %
Clinical Associate (LPN, medical assistant)	24.2 %
Administrative Clerk	12.3 %
Clinic characteristics (respondent-level)	
Clinic workload and capacity	
PCPs with panels over-capacity (proportion)	31.4 %
Mean level of over-capacity for these panels	13.3 %
Adjusted panel size (mean number of patients)	1164
Patient intensity (mean DCG patient average)	0.60
Annual primary care visits	36,747
Hospital-based clinic (proportion)	38.7 %
Academic-affiliation agreement	34.5 %

Comparing the clinics in our sample with clinics excluded due to lack of survey data, the clinics that lack survey data had significantly smaller overall patient panels, lower annual visits, and were more likely to be community-based rather than hospital-based outpatient clinics (Table 2). These clinics were no different in terms of average panel size (i.e., the average number of patients cared for by a given provider), average patient comorbidities, or the proportion of providers with panels that were overcapacity. The proportion of clinics excluded for lack of survey data differed by Pi^2^ group. For high and middle Pi^2^ clinics, 70 and 73 % had survey data, respectively. Whereas for low Pi^2^ clinics, 48 % had survey data. The facilitators that were most frequently available were teamlet huddles (90.7 %), measurement tools (87.8 %), and local education sessions (87.7 %), while the least frequently available were quality improvement methods (58.9 %) and the VHA PCMH toolkit (70.6 %) (Table 3). The facilitators most often rated as very helpful were teamlet huddles (53.4 %), regular team meetings (44.7 %), and disease registries (32.9 %) (Table 4). The barriers to PCMH implementation most frequently reported as limiting delivery of patient-centered care were difficulty recruiting and retaining providers (59.5 %), volume of automated clinical reminders (54.0 %), and difficulty recruiting and retaining non-provider clinicians (49.5 %) (Table 5).

**Table 2:** Comparison of clinics with and without survey data stratified by Pi^2^ category

	Highest Pi^2^ clinics			Middle Pi^2^ clinics			Lowest Pi^2^ clinics		
	Surveys	No surveys	*p* value	Surveys	No surveys	*p* value	Surveys	No surveys	*p* value
Number	54	23	–	547	202	–	42	45	–
% of Pi2 group	70.1 %	29.9 %	–	73.0 %	27.0 %	–	48.3 %	51.7 %	–
Mean total patients	3210.9	1914.5	<0.001	7148.2	3397.0	<0.001	7709.6	2875.4	<0.001
Mean total annual visits	12,034.4	7613.0	<0.001	24,851.3	11,815.7	<0.001	24,668.5	9903.6	<0.001
Adjusted panel size (mean number of patients)	1130.9	1096.9	0.68	1117.2	1130.1	0.57	1241.6	1328.9	0.23
Average DCG of panel	0.562	0.537	0.55	0.588	0.537	0.0002	0.531	0.497	0.10
PCPs with panels over-capacity (proportion)	25.93 %	13.04 %	0.22	25.51 %	20.00 %	0.13	33.33 %	35.90 %	0.81
VAMC (proportion)	7.41 %	0.00 %	0.99*	23.20 %	5.13 %	<0.001	22.50 %	0.00 %	0.99*

*p value for this comparison may not be reliable due to there being no VAMCs in the No Surveys group

**Table 3:** Odds of a respondent’s clinic having a high-Pi^2^ (vs. low-Pi^2^) score as a function of reporting facilitators were available or that respondents were involved in facilitator activities

		ICC		High Pi^2^ vs. low Pi^2^	
	%^a^	Clinic	Occup.	Odds ratio	95 % CI^b^
Local education session	87.7	0.15	0.02	1.50	(0.77, 2.93)
Learning collaborative	80.0	0.12	0.01	1.20	(0.67, 2.14)
Measurement tools	87.8	0.14	0.05	*3.47*	(*1.47*, *8.16*)
Teamlet huddles	90.7	0.24	0.06	*3.91*	(*1.34*, *11.44*)
Regular team meetings	84.5	0.19	0.02	*2.88*	(*1.31*, *6.30*)
Information systems	83.3	0.18	0.02	*2.42*	(*1.21*, *4.82*)
New approaches to scheduling	80.9	0.12	0.01	1.34	(0.76, 2.34)
Quality improvement methods	58.9	0.07	0.01	1.38	(0.85, 2.25)
Disease registries	73.1	0.13	0.04	*2.01*	(*1.11*, *3.65*)
PACT toolkit	70.6	0.11	0.01	1.57	(0.88, 2.80)

^a^The overall percentage of respondents who reported the facilitator was available or that the respondent was involved in facilitator activities

^b^Odds ratios with 95 % confidence intervals that do not cross 1.0 are in italics

**Table 4:** Odds of a respondent’s clinic having a high-Pi^2^ (vs. low-Pi^2^) score as a function of reporting facilitators were very helpful for implementation of PACT

		ICC		High Pi^2^ vs. low Pi^2^	
	%^a^	Clinic	Occup.	Odds ratio	95 % CI^b^
Local education session	25.7	0.09	0.04	*2.02*	(*1.13*, *3.63*)
Learning collaborative	26.0	0.06	0.02	1.50	(0.87, 2.59)
Measurement tools	25.2	0.10	0.04	1.44	(0.85, 2.43)
Teamlet huddles	53.4	0.11	0.01	1.57	(0.94, 2.61)
Regular team meetings	44.7	0.07	0.01	*1.68*	(*1.05*, *2.70*)
Information systems	30.4	0.08	0.02	*1.97*	(*1.19*, *3.24*)
New approaches to scheduling	29.3	0.05	0.01	1.70	(1.00, 2.89)
Quality improvement methods	23.5	0.06	0.03	2.00	(0.93, 4.31)
Disease registries	32.9	0.09	0.01	*1.90*	(*1.22*, *2.96*)
PACT toolkit	24.7	0.09	0.04	1.59	(0.85, 2.97)

^a^The overall percentage of respondents who reported the facilitator was very helpful for implementation of PACT

^b^Odds ratios with 95 % confidence intervals that do not cross 1.0 are in italics

**Table 5:** Odds of a respondent’s clinic having a low-Pi^2^ (vs. high-Pi^2^) score as a function of reporting that barriers limited delivery of optimal patient-centered care “a great deal”

		ICC		Low Pi^2^ vs. high Pi^2^	
	%^a^	Clinic	Occup.	Odds ratio	95 % CI^b^
Lack of support from clinical leadership	34.3	0.10	0.01	*2.15*	(*1.21*, *3.82*)
Difficulty accessing specialist care	43.1	0.14	0.01	1.52	(0.87, 2.66)
Poor communication with specialists within VA	38.7	0.13	0.02	1.26	(0.68, 2.35)
Poor communication with specialists outside the VA	35.2	0.05	0.00	1.47	(0.93, 2.31)
Poor communication around inpatient care	25.1	0.08	0.01	1.21	(0.59, 2.47
Lack of control over my schedule	34.7	0.10	0.03	*1.98*	(*1.15*, *3.43*)
Lack of responsiveness to my requests for assistance from my team members	24.0	0.06	0.01	1.65	(0.99, 2.78)
Inadequate time allotted to provide counseling or education	36.9	0.05	0.01	1.24	(0.79, 1.95)
Inadequate time allotted to provide follow-up care	37.3	0.09	0.01	1.59	(0.97, 2.61)
Patients have limited VA benefits	24.2	0.03	0.02	1.59	(0.95, 2.67)
Preferred medications are difficult to obtain	28.4	0.10	0.00	1.21	(0.74, 2.00)
Inadequate support for patient behavioral change needs	26.4	0.09	0.00	*2.15*	(*1.36*, *3.38*)
Recruiting and retaining providers	59.5	0.27	0.00	*2.37*	(*1.29*, *4.33*)
Recruiting and retaining non-provider clinicians	49.5	0.16	0.00	*2.17*	(*1.17*, *4.00*)
Recruiting and retaining non-clinicians	43.0	0.10	0.01	*1.96*	(*1.09*, *3.53*)
Clinical reminder volume	54.0	0.08	0.05	1.31	(0.75, 2.27)
Delivering opiate therapy	38.9	0.08	0.04	1.55	(0.84, 2.86)
Time & effort to input notes	35.2	0.05	0.04	1.16	(0.66, 2.05)
High volume of Computerized Patient Record System (CPRS) alerts	41.5	0.08	0.05	1.48	(0.79, 2.77)

^a^The overall percentage of respondents who reported the barrier limited delivery of optimal patient-centered care a great deal

^b^Odds ratios with 95 % confidence intervals that do not cross 1.0 are in italics

When we assessed the level of concordance within the clinic site, for facilitator availability, the median ICC was 0.14 and ranged from a low of 0.07 (quality improvement methods) to a high of 0.24 (teamlet huddles) (Table 3). For facilitator helpfulness, the median ICC was 0.08 and ranged from a low of 0.05 (new approaches to scheduling) to a high of 0.11 (teamlet huddles) (Table 4). For barriers, the median ICC was 0.09 and ranged from a low of 0.03 (patients have limited VAVHA benefits) to a high of 0.27 (recruiting and retaining providers) (Table 5).

When we assessed the level of concordance within occupation, for facilitator availability, the median ICC was 0.02 and ranged from a low of 0.01 (3 facilitators) to a high of 0.06 (teamlet huddles) (Table 3). For facilitator helpfulness, the median ICC was 0.02 and ranged from a low of 0.01 (4 facilitators) to a high of 0.04 (PACT toolkit and measurement tools) (Table 4). For barriers, the median ICC was 0.01 and ranged from a low of 0.00 (5 barriers) to a high of 0.05 (clinical reminder volume and delivering opiate therapy) (Table 5).

### Facilitators, adjusted analyses

The availability of five facilitators was associated with higher odds of a respondent’s clinic being in the highest versus lowest decile for Pi^2^ scores (Table 3): teamlet huddles (OR = 3.91, CI 1.34–11.44), measurement tools (OR = 3.47, CI 1.47–8.16), regular team meetings (OR = 2.88, CI 1.31–6.30), information systems (OR = 2.42, CI 1.21–4.82), and disease registries (OR = 2.01, CI 1.11–2.80).

The helpfulness of four facilitators was associated with higher odds of a respondent’s clinic being in the highest versus lowest decile for Pi^2^ scores (Table 4): local education sessions (OR = 2.02, CI 1.13–3.63), information systems (OR = 1.97, CI 1.19–3.24), disease registries (OR = 1.90, CI 1.22–2.96), and regular team meetings (OR = 1.68, CI 1.05–2.70).

### Barriers, adjusted analyses

For six of the 19 barrier items, reporting that delivery of patient-centered care was limited a great deal by the barrier was associated with higher odds of a respondent’s clinic being in the lowest versus highest decile for Pi^2^ scores (Table 5): difficulty recruiting and retaining providers (OR = 2.37, CI 1.29–4.33); difficulty recruiting and retaining non-provider clinicians (OR = 2.17, CI 1.17–4.00); lack of support from clinical leadership (OR = 2.15, CI 1.21–3.82); lack of control over one’s schedule (OR = 1.98, CI 1.15–3.43); inadequate support for patient behavioral change needs (OR = 2.15, CI 1.36–3.38); and recruiting and retaining non-clinicians (OR = 1.96, CI 1.09–3.53).

### Medium Pi^2^-score clinics versus low

We also found that the availability of three facilitators was associated with higher odds of the respondent’s clinic being in the middle eight deciles versus the lowest, and that the reporting of seven barriers was associated with higher odds of the respondent’s clinic being in the lowest Pi^2^ decile versus the middle eight deciles (Appendix 1). In all cases, these were also significant associations in the comparison of respondents in clinics with the highest versus lowest decile for Pi^2^ score, and in all cases, the associations were smaller but in the expected direction. There were no significant associations between helpfulness of facilitators and the odds of a respondent’s clinic being in the middle eight deciles for Pi^2^ scores versus in the lowest decile.

In the secondary analyses testing how robust the results were to different assumptions about NA/Don’t Know responses, the results were virtually identical.

## Discussion

To our knowledge, ours is the first paper to explore the associations of specific PCMH facilitators and barriers to patient-centered care with a validated measure of PCMH implementation across a population of clinics all implementing a PCMH model. This is important because outcomes, such as patient experience and quality of care, differ significantly among clinics depending on how well a PCMH model is implemented [13, 15].

We found that a range of facilitators and barriers were significantly associated with a validated measure of PCMH implementation. The relationships were all in the expected direction, and were frequently large. Respondents within clinics exhibited what organizational scholars generally consider modest (0.08 to 0.12) to high (>0.20) concordance on most items [16], which increases our confidence that surveys accurately captured clinic differences in barriers and facilitators.

Both the availability and ratings of facilitators as very helpful were associated with greater odds of a respondent’s clinic being in the highest versus lowest decile for Pi^2^ score. This suggests that, in 2012, primary care employees at the clinics with the most limited progress on VHA PCMH implementation may have lacked both access to or awareness of resources and may have found that available implementation resources failed to meet their needs.

The facilitators that distinguished high-implementation from low-implementation clinics included factors related to infrastructure, such as disease registries and recruiting and retaining employees, as well as factors related to process, such as reported helpfulness of teamlet huddles and team meetings, and local PCMH education sessions.

These national survey results corroborate findings from prior in-depth evaluations of PCMH implementation at VHA demonstration clinics. At demonstration clinics, data infrastructure was critical for practice redesign to be successful, and to allow for effective panel management [9], and VHA providers who participated in daily huddles reported greater self-efficacy for implementing PCMH changes [17].

Recruiting and retaining providers and non-provider clinicians were both among the most prevalent barriers, and had the strongest associations with PCMH implementation. Respondents at clinics with the lowest Pi^2^ scores were more likely to report patient-centered care was hindered due to challenges in recruiting and retaining providers, non-provider clinicians and non-clinicians. This is consistent with earlier findings that inadequate staffing of teamlets was a major challenge to making the VHA PCMH model work [18], and that provider turnover had worsened since the launch of the PCMH initiative, with turnover significantly greater for older and more experienced providers [19]. Turnover in PCMH teamlet members likely contributes to other barriers (e.g., difficulties in holding meetings regularly to coordinate work, and lack of responsiveness from team members), which may make turnover among both providers and other team members a particularly important challenge to effectively implementing the new model.

Although clinical reminder volume and electronic medical record system alert volumes were frequently rated by respondents as barriers to delivery of patient-centered care, they were not associated with the odds of a respondent’s clinic being in the highest versus lowest decile for Pi^2^ score. Neither was delivery of opiate therapy which was cited as a major concern in open-text responses from this same survey [18]. A probable explanation is that these barriers were pervasive system-wide, and exhibit limited variation across clinics, and therefore do not differentiate PCMH implementation among clinics. However, due to their prevalence, these barriers may be very important to address, irrespective of whether they are associated with PCMH implementation.

### Limitations

This was a cross-sectional analysis of observational data. This design is susceptible to unobserved confounding, and we cannot assume that the observed associations are causal. We found potential selection bias in terms of the clinics excluded due to lack of survey data. These clinics tended to be smaller, community-based clinics, and were more likely to be a low-Pi^2^ clinic. This type of selection bias would most likely increase our risk of a type 2 error, and it is possible that we would have found additional significant associations with a more representative sample of clinics. It is also possible that our findings are due to inferential bias, in which respondents at high-implementation clinics infer that they encountered fewer barriers and received more helpful resources because they observed greater implementation of the VHA PCMH model. However, this type of bias, called representativeness heuristic, usually arises when someone infers an answer to a more complex question (e.g., how much progress have you made on VHA PCMH) from a simpler, specific observation (e.g., have you been able to hire staff), which is the opposite of the present analysis [20]. It is also of greater concern when the respondent is rating both the outcome and the explanatory variables. In this case, the outcome is a clinic-level index incorporating data from a range of sources and respondents completed the survey without knowledge of their clinic’s Pi^2^ score.

We tested 39 associations, and it is possible that significant findings are just chance associations. However, we found a total of 15 significant associations when we would expect to find approximately two by chance (39 * 0.05 = 1.95). Moreover, the associations were all in the expected direction, and we found a similar but weaker pattern of associations when comparing clinics in the lowest decile for Pi^2^ score with clinics in the middle eight deciles.

Finally, we used a discrete set of structured measures of barriers and facilitators of PCMH implementation that may have omitted important factors. Some factors we may have omitted, such as strategies for integrating additional clinic members, for example, pharmacists [21], could play an important role in implementation of the VHA PCMH model. Other potentially omitted factors, such as the effects of healthcare reform and conflicting criteria for PCMH designation [22], are likely less salient for VHA clinics, but may be vitally important in other settings.

## Conclusions

Overall, our findings suggest that a number of factors may have important influences on PCMH implementation, including structural factors, such as clinical IT and recruitment and retention of clinicians and staff, as well as process issues, such as team huddles and quality of local education sessions on PCMH.

Our findings make a modest but important contribution to the literature by exploring associations of key barriers and facilitators with PCMH implementation in a large, national sample of VHA primary care clinics using a validated, structured measure of PCMH implementation. These findings may help guide future research and may be useful to healthcare policy makers and leaders engaged in PCMH implementation, who must decide where to focus finite attention and devote limited resources.

## Appendix 1

**Table 6:** Odds of a respondent’s clinic being in the middle eight deciles for Pi^2^ score (vs. lowest decile) as a function of reporting facilitators were available or that respondents were involved in facilitator activities

	Medium Pi^2^ vs. low Pi^2^	
	Odds ratio	*p* value
Local education session	0.93	0.68
Learning collaborative	0.99	0.93
Measurement tools	*1.44*	*0.03*
Teamlet huddles	1.36	0.15
Regular team meetings	1.27	0.14
Information systems	1.35	0.06
New approaches to scheduling	1.08	0.66
Quality improvement methods	1.24	0.16
Disease registries	*1.60*	*0.02*
PACT toolkit	1.01	0.96

## Appendix 2

**Table 7:** Odds of a respondent’s clinic being in the middle eight deciles for Pi^2^ score (vs. lowest decile) as a function of reporting facilitators were very helpful for implementation of PACT

	Medium Pi^2^ vs. low Pi^2^	
	Odds ratio	*p* value
Local education session	1.25	0.26
Learning collaborative	1.17	0.43
Measurement tools	1.14	0.51
Teamlet huddles	1.12	0.46
Regular team meetings	1.30	0.10
Information systems	1.32	0.08
New approaches to scheduling	1.17	0.38
Quality improvement methods	1.13	0.64
Disease registries	1.30	0.09
PACT toolkit	0.98	0.92

## Appendix 3

**Table 8:** Odds of a respondent’s clinic being in the lowest decile for Pi^2^ score (vs. middle eight deciles) as a function of reporting that barriers limited delivery of optimal patient-centered care a great deal

	Low Pi^2^ vs. medium Pi^2^	
	Odds ratio	*p* value
Lack of support from clinical leadership	1.34	0.10
Difficulty accessing specialist care	1.27	0.25
Poor communication with specialists within VA	1.04	0.84
Poor communication with specialists outside the VA	1.01	0.92
Poor communication around inpatient care	1.14	0.46
Lack of control over my schedule	*1.37*	*0.04*
Lack of responsiveness to my requests for assistance from my team members	*1.58*	*0.01*
Inadequate time allotted to provide counseling or education	1.01	0.95
Inadequate time allotted to provide follow-up care	1.22	0.30
Patients have limited VA benefits	1.42	0.05
Preferred medications are difficult to obtain	1.18	0.36
Inadequate support for patient behavioral change needs	*1.40*	*0.03*
Recruiting and retaining providers	*1.56*	*0.05*
Recruiting and retaining non-provider clinicians	*1.69*	*0.004*
Recruiting and retaining non-clinicians	1.38	0.06
Clinical reminder volume	1.15	0.39
Delivering opiate therapy	1.08	0.65
Time & effort to input notes	1.02	0.93
CPRS alerts volume	1.24	0.20
